# Metformin modulates mitochondrial metabolism and epigenetic dysregulation in *DNMT3A*-mutated clones

**DOI:** 10.1186/s43556-025-00311-5

**Published:** 2025-09-12

**Authors:** Xiaoyin Hu, Bin Wang, Long Zhang

**Affiliations:** 1 Department of Radiation Oncology and the State Key Laboratory of Transvascular Implantation Devices, School of Medicine, the Second Affiliated Hospital, Life Sciences Institute, Zhejiang University, Hangzhou, China; 2 Department of Infectious Diseases, Children’s Hospital Affiliated to Soochow University, Soochow University, Suzhou, China; 3 Frontiers Medical Center, Tianfu Jincheng Laboratory, Chengdu, China

In a recent study published in *Nature*, Mohsen et al*. *[1] revealed that *DNMT3A* R882-mutated clonal haematopoiesis (CH) relies on heightened mitochondrial respiration in haematopoietic stem and progenitor cells (HSPCs). They further showed that the anti-diabetic drug metformin can suppress the competitive advantage of mutant HSPCs by targeting this metabolic reprogramming and restoring normal epigenetic regulation. These results suggest metformin could potentially prevent CH-related disorders in *DNMT3A* R882 mutation carriers.

CH occurs as a haematopoietic stem cell (HSC) acquires a mutation that provides a competitive advantage over wild-type HSCs, leading to clonal expansion. This process is linked to an increased risk of developing haematologic malignancies and age-related inflammatory diseases [2]. Even though it's a big deal clinically, there aren't any proven treatments to stop mutant HSCs from growing. DNMT3A (DNA Methyltransferase 3 A), which is important for adding methyl groups to DNA, is one of the most commonly mutated genes in CH [3]. The R882 mutation in this gene is especially common. The mechanisms through which *DNMT3A* R882 mutations promote clonal expansion, and how this process might be targeted therapeutically, remain unclear.

This study looked into how the *Dnmt3a* R878H mutation (analogous to the human *DNMT3A* R882H) affects mouse HSPCs. The authors demonstrated that mutant HSPCs have higher mitochondrial activity compared to normal cells, and this change in metabolism gives them a competitive edge. To explore potential treatments, they tested metformin, a common diabetes drug known to inhibit mitochondrial function. Treatment with metformin considerably slowed down the growth of mutant HSPCs both in vitro and in vivo, suggesting that targeting mitochondrial activity might be a promising way to combat CH.

To understand how metformin works at the molecular level, a multi-omics approach was employed. They found that metformin boosts the ability of mutant HSPCs to modify DNA and histones by increasing the activity of DNMT3A and the Polycomb Repressive Complex 2 (PRC2), which catalyzes histone H3 lysine 27 trimethylation. This helped restore the normal patterns of DNA and histone modifications that were messed up by *DNMT3A* R882 mutation. Importantly, these effects mainly affected the mutant cells with little impact on normal cells, indicating metformin’s potential as a targeted treatment for CH caused by the mutation.

To confirm these results in human cells, they used two different approaches. First, they used RNA interference to reduce *DNMT3A* expression in human HSPCs, mimicking the mutation’s effect. Just like in the mouse study, metformin treatment mitigated the enhanced fitness of these knockdown cells. Second, they created a precise gene editing method to insert the *DNMT3A* R882H mutation into human HSPCs efficiently. This safer editing technique is especially useful for studying cell functions without causing extra damage. Notably, metformin also reduced the growth advantage of the edited mutant human cells, supporting its potential real-world application.

The study also looked at what these findings could mean for future treatments. CH is linked not just to blood cancers but also to age-related inflammation. Interventions capable of suppressing mutant clone expansion could therefore substantially improve health outcomes in aging populations. Given its safety profile, affordable, and widespread clinical use, metformin emerges as a promising candidate for repurposing in CH prevention [4]. In addition, the authors proposed that other strategies targeting one-carbon metabolism, such as folic acid supplementation, may help restore epigenetic regulation and constrain clonal proliferation. Together, these results offer a compelling preclinical foundation for future clinical trials evaluating metformin and similar approaches in patients with *DNMT3A* R882-mutated CH.

These findings also prompt several key questions for future investigation. Although metformin demonstrated selectivity for mutant HSPCs, its long-term impact on normal haematopoietic stem cells and other tissues requires further evaluation. Moreover, while the study centered on *DNMT3A* R882 mutations, CH can also be driven by diverse mutations in genes such as *TET2* (ten-eleven translocation 2) and *ASXL1* (additional sex combs like 1). It remains uncertain whether metformin or related metabolic therapies would prove beneficial in non-*DNMT3A*-driven CH. Lastly, as this research was performed in preclinical models, its clinical translation will need to carefully address optimal dosing, treatment duration, and appropriate patient selection.

In a complementary study, Gozdecka et al*. *[5] conducted a genome-wide CRISPR screen on primary mouse *Dnmt3a*^R882H/+^ HSPCs to identify genes specifically vulnerable in *Dnmt3a* R882-mutant cells. Their results indicated that mutant HSPCs exhibit increased reliance on mitochondrial metabolism, particularly oxidative phosphorylation. Subsequent metabolic flux analysis confirmed this increased reliance on oxidative phosphorylation as a distinguishing metabolic feature of mutant cells. Gozdecka et al*.* then focused on mitochondrial metabolism inhibitors, including metformin, and found that metformin selectively curbed clonal expansion of *Dnmt3a* R882-mutant HSPCs in vivo. This effect was validated in competitive transplantation assays, where metformin-treated mutant HSPCs showed reduced engraftment and clonal expansion compared to untreated controls, with minimal impact on wild-type cells. To underscore translational potential, Gozdecka et al*.* tested metformin on human *DNMT3A* R882-mutant CH samples and demonstrated similar suppression of clonal expansion. Furthermore, by analyzing data from the UK Biobank, they found that metformin use was correlated with a lower prevalence of *DNMT3A* R882-mutant CH, even after accounting for confounders such as diabetes and body mass index.

Both studies consistently underscore the critical role of mitochondrial metabolism in *DNMT3A* R882-mutant CH and highlight the therapeutic potential of targeting this pathway. While Mohsen et al*.* focused on the epigenetic mechanisms underlying metformin's effects, Gozdecka et al*.* provided population-level evidence supporting the clinical relevance of metformin in suppressing *DNMT3A* R882-mutant CH. Collectively, these findings emphasize the promise of targeting metabolic vulnerabilities in *DNMT3A* R882-mutant HSPCs as a novel strategy for preventing CH-associated diseases. However, although Gozdecka et al*.* analyzed data from the UK Biobank, which supports their conclusions, it is important to note that both studies primarily demonstrated the efficacy of metformin using a mouse model and rely on in vitro models for human HSPCs. Robust humanized in vivo models that faithfully mimic human CH remain scarce. Developing such models—potentially through advanced xenograft techniques or the engineering of humanized hematopoietic niches—will be crucial for validating the therapeutic effects of metformin and accelerating its clinical translation.

Overall, these findings provide a strong foundation for future clinical trials investigating metformin and other mitochondrial metabolism inhibitors in patients with *DNMT3A* R882-mutated CH. Moreover, uncovering other metabolic vulnerabilities in *DNMT3A* R882-mutant cells could enable combination strategies that improve efficacy while reducing off-target effects. Moving forward, subsequent studies should focus on optimizing metformin dosing, assessing long-term safety, and establishing biomarkers for patient selection. Despite these challenges, the current work marks considerable progress toward preventing CH, holding promise for improving health outcomes in affected individuals globally (Fig. 1).

**Fig. 1:**
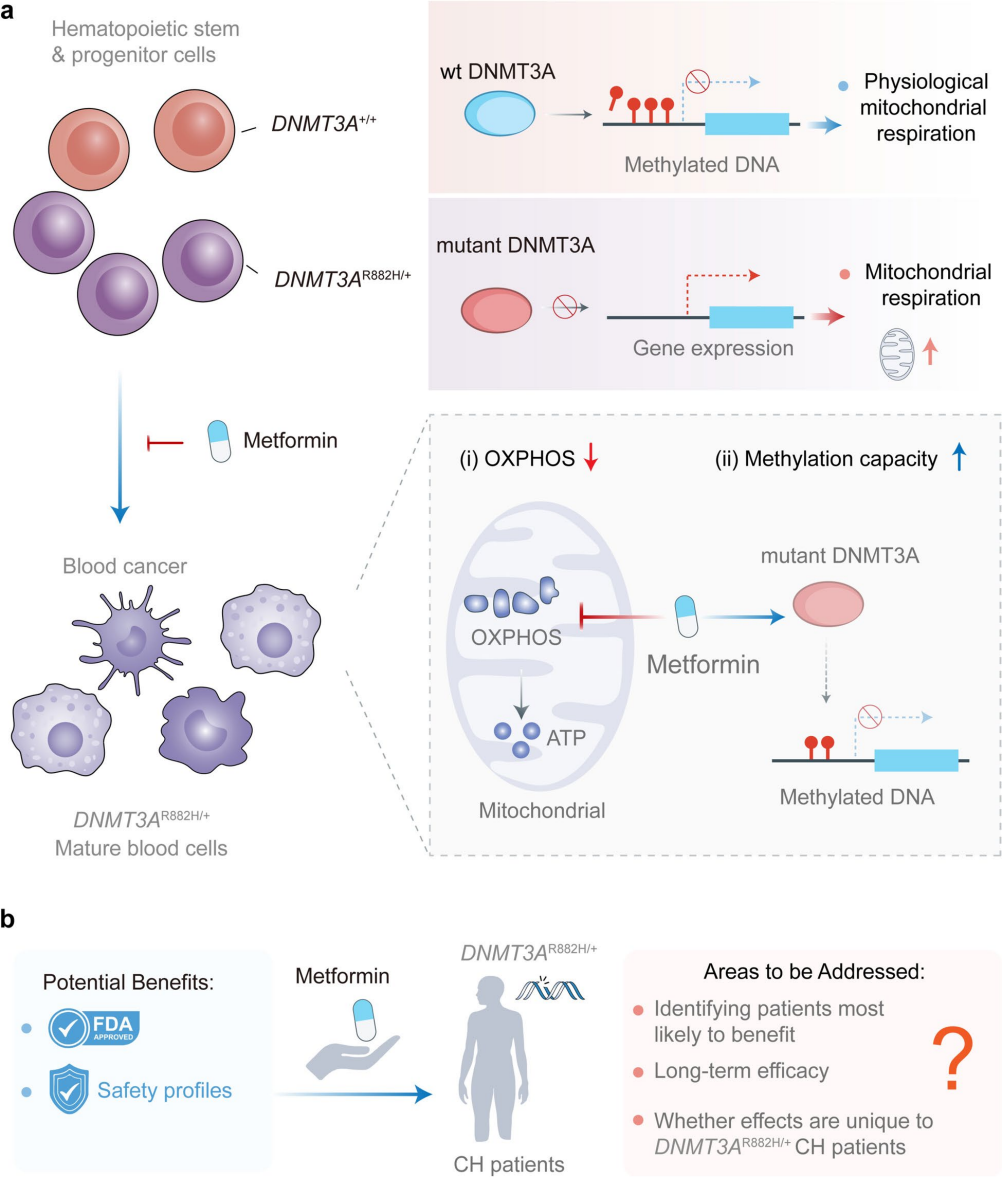
Counteracting clonal hematopoiesis. **a** Hematopoietic stem and progenitor cells (HSPCs) generate all blood cell types throughout life. With aging, clonal hematopoiesis can emerge, where cells with advantageous *DNMT3A* (DNA methyltransferase 3A) mutations dominate blood cell production, potentially leading to conditions like blood cancers. *DNMT3A* normally adds methyl groups to DNA to repress gene transcription. *DNMT3A*-mutated cells exhibit heightened mitochondrial respiratory capacity, creating a metabolic vulnerability exploitable for therapeutic intervention. The anti-diabetic drug metformin inhibits oxidative phosphorylation, reducing the survival of *DNMT3A*-mutant cells without affecting healthy cells, offering a potential strategy to counteract clonal hematopoiesis. **b** Summary of potential benefits and risk stratification of Metformin in the treatment *DNMT3A* R882-mutant HSPCs. OXPHOS: Oxidative Phosphorylation

## Data Availability

Not applicable.
